# How Vine Shoots as Fillers Impact the Biodegradation of PHBV-Based Composites

**DOI:** 10.3390/ijms21010228

**Published:** 2019-12-28

**Authors:** Grégoire David, Julie Michel, Emmanuelle Gastaldi, Nathalie Gontard, Hélène Angellier-Coussy

**Affiliations:** JRU IATE 1208—CIRAD/INRA/Montpellier Supagro/University of Montpellier, 2 Place Pierre Viala, Bat 31, CEDEX 01, 34060 Montpellier, France; gregoire.david@supagro.fr (G.D.); julie.michel@supagro.fr (J.M.); emmanuelle.gastaldi@umontpellier.fr (E.G.); nathalie.gontard@inra.fr (N.G.)

**Keywords:** biocomposites, natural fibers, biodegradation, poly(3-hydroxybutyrate-3-hydroxyvalerate), vine shoots, polyphenols extraction

## Abstract

Vine shoots are lignocellulosic agricultural residues. In addition to being an interesting source of polyphenols, they can be used as fillers in a poly(3-hydroxybutyrate-3-hydroxyvalerate) (PHBV) matrix to decrease the overall cost and to propose an alternative to non-biodegradable fossil-based materials. The objective of the present work was to investigate how the incorporation of vine shoots fillers and a preliminary polyphenol extraction step could impact the biodegradability of biocomposites. Biocomposites (20 wt %) were produced by microcompounding. The biodegradation of materials was assessed by respirometric tests in soil. The negative impact of polyphenols on the biodegradability of vine shoots was confirmed. This was supported by crystallinity measurements and scanning electron microscopy (SEM) observations, which showed no difference in structure nor morphology between virgin and exhausted vine shoots particles. The incorporation of vine shoots fillers in PHBV slightly accelerated the overall biodegradation kinetics. All the biocomposites produced were considered fully biodegradable according to the French and European standard NF EN 17033, allowing the conclusion that up-cycling vine shoots for the production of lignocellulosic fillers is a promising strategy to provide biodegradable materials in natural conditions. Moreover, in a biorefinery context, polyphenol extraction from vine shoots has the advantage of improving their biodegradability.

## 1. Introduction

The demand for plastics is continually increasing, reaching 350 million tons in 2018, while it was 250 million tons in 2010 [1]. The packaging sector stands for the plastics’ largest market, with 40% of the overall plastic demand in Europe. A global shift from reusable to single-use containers has accelerated the growth of plastic consumption. Most of them are materials designed for immediate disposal. Geyer et al. [2] estimated that 60% of all plastics ever produced have been discarded and are accumulating in landfills or in the natural environment. Today, 32% of plastic packaging leaks out of collection systems worldwide of which at least 8 million tons end up in the ocean [3,4]. To address the overwhelming negative environmental issues of plastic packaging and to enter the virtuous loop of a circular economy, it is urgent and crucial to mitigate the negative burden of packaging resources (renewable without competition with food resources versus oil-based) and waste management (fully biodegradable in natural conditions versus accumulation). The main driver of biodegradable plastics is that biodegradability offers a closed loop value chain and avoids the persistence of micro-plastics in our environment.

Biodegradable plastics have raised great interest, especially for packaging and agriculture sectors, which usually have short-term and single use applications. Polyhydroxyalkanoates (PHA) are a class of polyesters that are synthesized by bacteria grown on carbon sources, stemming from renewable resources and even organic residues. Up to now, the global production of PHAs has represented 3.2% of all the biopolymers after starch blends, polylactic acid (PLA), and polybutylene adipate terephthalate (PBAT) [5]. They are attracting attention from both academia and industry due to their full biodegradability in natural conditions, even in marine conditions, and the possibility of synthetizing a large range of copolymers with different properties. Among PHAs, poly(3-hydroxybutyrate-3-hydroxyvalerate) (PHBV) is a short-chain length copolymer, which is deeply studied and already available on the market. Depending on its hydroxyvalerate (HV) content, mechanical properties are close to those of common polyolefins, with interesting oxygen and water vapor transfer properties, which makes it a good candidate for packaging applications [6]. The biodegradation behavior of PHBV at laboratory scale has been well investigated [7,8]. It can be degraded readily in natural conditions (soil [9], marine waters [10]), producing monomers that are further metabolized [11]. The biodegradation of PHAs happens by the breakage of the ester from the end of the chain. PHAs are fully biodegradable in natural conditions, whereas PLA is only biodegradable in industrial conditions. Poly(3-hydroxybutyrate) (PHB) can be considered as a reference material, instead of cellulose, in the standards of biodegradation for soil and water environments, respectively NF EN 17033 and ISO DIS 14852. Increasing the HV content is one of the strategies to improve the biodegradability [12] in addition to get better functional properties: lower melting temperature and higher elongation at break [13].

The vine industry is a very important economic activity that generates vast amounts of agricultural residues, particularly vine shoots (ViSh). On average, 2 tons of dry shoots are obtained per hectare every year [14]. Vine shoots correspond to the woody part obtained after pruning and are one of the most important primary solid lignocellulosic residues in agriculture. Depending on the region, ViSh are either collected and burnt or left on the vineyards where they could be roughly grounded and used as soil amendment. Their current economic value is therefore very small and better options for management are awaited. Several studies have reported that ViSh could be an interesting source of high value added molecules, such as polyphenols, that are used in the pharmaceutical and cosmetic sectors due to their antioxidant properties [15,16,17]. Hence, in a biorefinery context, polyphenols could be first extracted from vine shoots, and the exhausted lignocellulosic residues could be further used as fillers in biocomposites [18]. The potential of using ViSh as fillers in a PHBV matrix has been shown [18,19]. It was concluded that such biocomposites can surely provide a sustainable alternative to fossil-based materials and a new way to manage agro-residues.

Generally, composite materials display a higher biodegradation rate than the virgin polymer matrix due to the higher water sorption of lignocellulosic fillers. This is the case with PHA-based composites filled with wood flour or olive pomace [20,21,22]. Recently, Chan et al. [23] investigated the role of wood flour in promoting biodegradation of macroscopic PHBV-based composite specimens in soil. The degradation rate of composites increased with wood content, and was five times greater for composites with 50 wt % of fillers than for neat PHBV. This was explained by the formation of cracks along the PHBV–wood interfaces significantly increasing the accessible surface area. However, in another study dealing with PHBV/wheat straw composites, the results were contrasting [24]. It has been reported that the presence of straw did not affect the biodegradation rate when it was evaluated in a liquid environment and in long-term soil burial tests. Moreover, in composting conditions, the rate of biodegradation was reduced for composites filled with more than 10 wt % of wheat straw. This negative effect was attributed to the fact that fungi, that degrade lignin, were not favored by the neutral pH encountered in compost medium. Several studies have shown that after removal of extractives, wood is more susceptible to degradation [25,26]. Nascimiento et al. [27] demonstrated the role of phenolic compounds in the natural resistance of wood biodegradation. Wood extractives such as flavonoids or stilbenes are even considered natural preservatives against wood decay organisms. 

In this context, the aim of the present study is to examine for the first time the impact of vine shoots on the aptitude of PHBV/ViSh composites to biodegrade in soil. This is a main feature for such materials that are destined for short-term applications, like packaging with trays or agriculture with pots. For that purpose, ViSh fillers are produced by dry milling. The impact of polyphenols extraction with a mixture of acetone/water (*v*/*v*, 75:25) on the biodegradation behavior of ViSh and resulting biocomposites is also investigated. PHBV/ViSh composites are prepared by microcompounding with a filler content of 20 wt %. The biodegradation behavior of both virgin and exhausted ViSh fillers and resulting composites is assessed through respirometric tests monitored in aerobic conditions. Results are discussed in relation to the composition and the structure of materials.

## 2. Results and Discussion

### 2.1. Biodegradability of Vine Shoots (ViSh) Fillers

**Figure 1:**
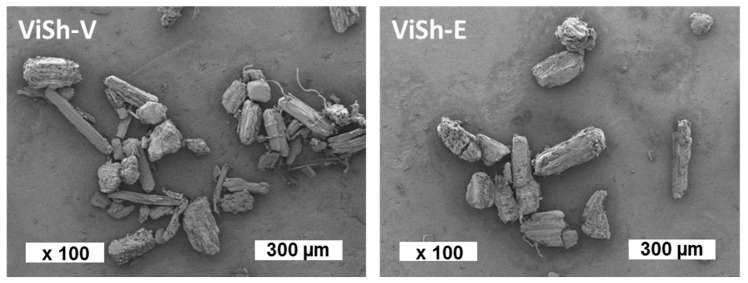
Scanning electron microscopy (SEM) pictures of vine shoots fillers.

Both fillers, i.e., virgin (ViSh-V) and exhausted (ViSh-E), were subjected to the same milling processes in order to get particles with the same size. It was verified by scanning electron microscopy (SEM) observations that both fillers did not display notable difference in terms of morphology and surface aspect (Figure 1). Laser diffraction analysis (Malvern Mastersizer 2000) allowed the measurement of their median apparent diameter, which was 143 ± 10 µm for the two fractions. Fillers were introduced in the soil medium without additional milling.

Table 1 presents the biochemical composition of the two ViSh fillers. It can be seen that the Klason lignin was slightly lower for ViSh-E. This was probably due to the removal of condensed tannins during extraction, that were first included in the Klason lignin in the case of ViSh-V. The ash content was slightly higher in the case of ViSh-E due to the removal of 10 wt % of the total mass during the extraction. As expected, the tannin content was reduced by 67% and resveratrol, a stilbenoid compound, was almost absent in ViSh-E (Table 1).

The protein content of ViSh-V fillers was slightly lower than values reported in the literature, with 3.3% instead of 5% [14]. After extraction, the protein content was reduced by half. The N content of ViSh particles was low, inducing a C/N ratio as high as 87 and 170, respectively, for ViSh-V and ViSh-E. A high C/N ratio was expected to induce a decrease in the biodegradability due to a surplus of degradable substrate [28].

**Figure 2:**
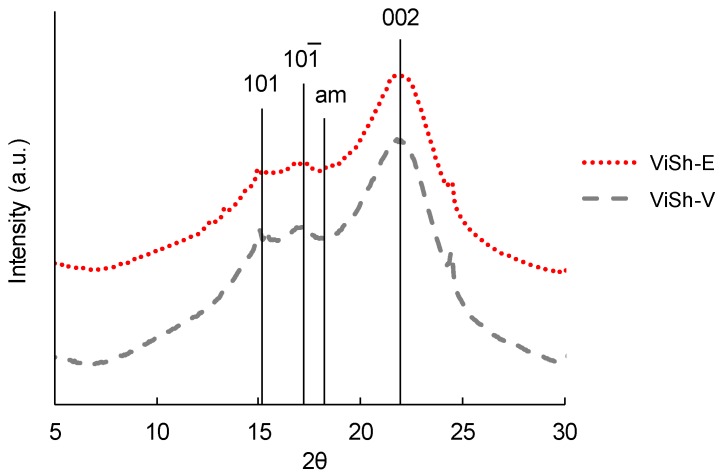
X-ray diffractograms of ViSh-V (virgin) and ViSh-E (exhausted), “am” stands for the amorphous scattering.

In order to analyze the crystallinity of the ViSh particles, X-ray diffraction (XRD) patterns were used (Figure 2). The crystallinity index (CrI) can be determined by using the following Equation (1) [29]:

(1) $$\mathrm{Crystallinity}\;\mathrm{index}\;\left(\mathrm{CrI}\right)=\;\frac{I_{002}-{\;I}_{\mathrm{am}}}{I_{002}},$$

where I_002_ is the peak intensity at 22°, representing the crystalline cellulose regions, and I_am_ is the intensity at 18°, representing the amorphous part.

The deduced crystallinity from the X-ray diffractograms was 30% and 32%, respectively, for ViSh-V and ViSh-E. It could be concluded that the acetone/water-based extraction treatment did not significantly impact the crystallinity of ViSh.

**Figure 3:**
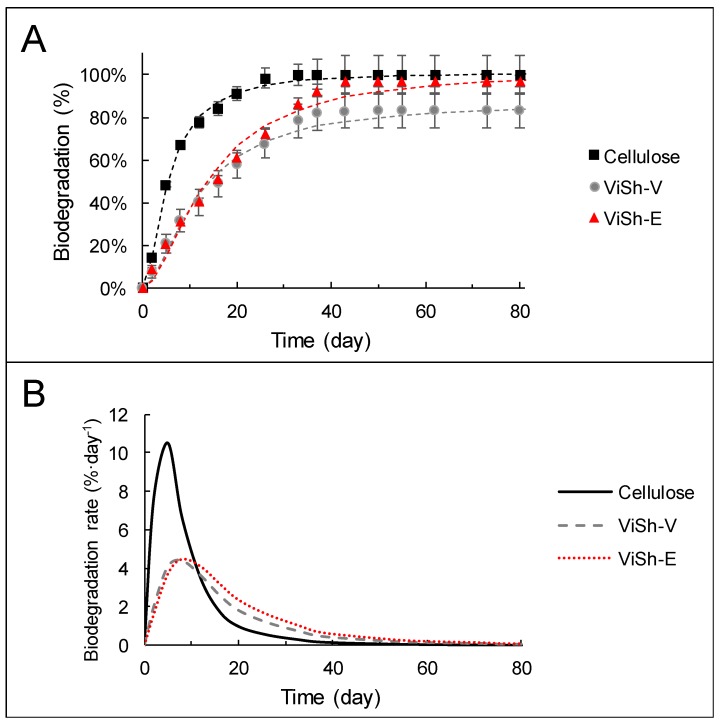
Kinetic of biodegradation (**A**) and biodegradation rate (**B**) of ViSh-fillers in soil.

The biodegradation rate of ViSh-V and ViSh-E fillers was assessed by monitoring the released carbon dioxide. The CO_2_ evolution is an indicator of the ultimate biodegradability resulting from mineralization of the organic carbon of the tested materials. The biodegradation curves of ViSh-V and ViSh-E presented in Figure 3 display a characteristic Hill sigmoidal shape with a low radius, close to 1. Generally, when microorganisms are involved, two phases are ascribed to the polymer degradation process. First, extracellular enzymes cleave the polymer chains into small fragments. Then, these fragments are mineralized into the cell and transformed into CO_2_, water minerals, and biomass [30]. Here, the two steps seemed to occur simultaneously.

Leaving aside standard requirements, the ViSh fillers biodegraded rather well compared to cellulose. The inherent structure and crystallinity of lignocellulose can be altered by milling. Mais et al. suggested that reducing the size of the sample by milling would make it more available for enzymatic degradation [31]. However, in the present case, the particle sizes were the same and the crystallinity indexes were very close, which would be expected not to induce a significant effect on the biodegradability.

Interestingly, the biodegradation curves showed that ViSh-V particles were not fully biodegradable because the entire material was not fully mineralized into CO_2_. The final biodegradation rate reached only 83.1%, which was below the standard of 90% (in absolute or relative to cellulose) to be considered as fully biodegradable as claimed by the NF EN 17033 standard. These results were not surprising, since lignocellulosic materials are complex materials in terms of structure and chemistry, usually resulting in quite a low biodegradation. Lignin is particularly recalcitrant to microbial degradation [32], fungi being the main lignin destroyers. It has been proven that the biodegradation level of biomass is higher for hemicellulose and cellulose than lignin [33]. Besides this, lignin is more hydrophobic than cellulose, as already shown using water contact angle measurements [19]. 

Moreover, as shown in Table 1, ViSh contains polyphenols, notably tannins, that are known to be recalcitrant to microbial attack [34,35]. They can even be toxic to some microorganisms, even if reports related to the ability of bacteria to degrade tannins are scarce. However, works have shown that fungi can degrade tannins, which can be useful to treat tannery effluents [35].

It can be noted that ViSh-E degraded better than ViSh-V. The exhausted ViSh had a final biodegradation level of 97%, allowing the conclusion that ViSh-E were biodegradable according to the NF EN 17033 standard. Such a behavior was mainly explained by the polyphenol removal during the extraction step.

It is worth noting that ViSh-V and ViSh-E started to degrade at the same rate and differentiated only after the 20th day. In addition, although ViSh-E reached a plateau at almost 100% of mineralization, its initial biodegradation rate was much lower than those of cellulose (Figure 3). This points out the fact that it is delicate to forecast the final biodegradation levels from the initial biodegradation rate. It is also the reason why the biodegradation curves obtained from the evolution of carbon dioxide release should reach a plateau phase, which reflects that no further biodegradation is expected. This is proof that the entire material was fully mineralized into carbon dioxide, attesting its final assimilation by microorganisms as requested in the majority of the standards dealing with the biodegradation assessment, and notably NF EN 17033. 

### 2.2. Biodegradability of PHBV-Based Composites

Composites were introduced in the soil after a milling and sieving step. Their median size determined by a laser diffraction particle size analyzer (Malvern Mastersizer 2000) was 350 ± 20 µm. 

**Figure 4:**
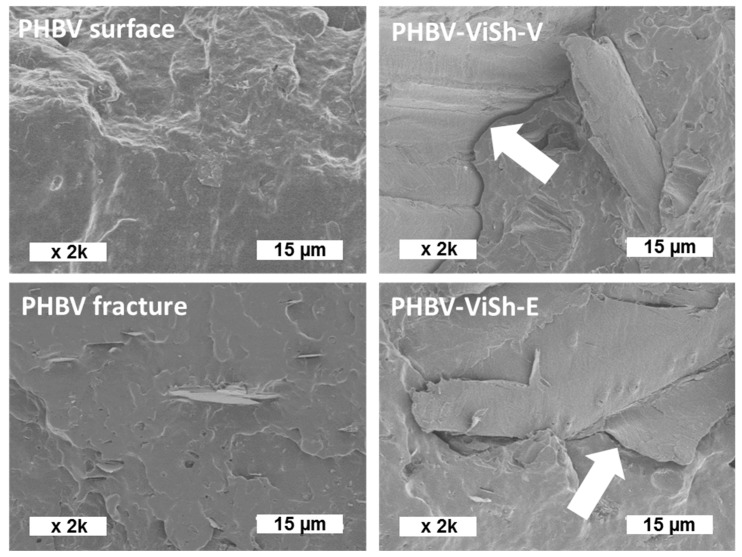
SEM pictures of the poly(3-hydroxybutyrate-3-hydroxyvalerate) (PHBV) surface and fracture (on the left) and SEM pictures of the PHBV-based composite fractures (filler content of 20 wt %) (on the right). White arrows show the matrix/filler interface.

Figure 4 presents the SEM pictures of PHBV and PHBV-based composite fractures before the biodegradation tests. The SEM image of the surface of PHBV film displayed a smooth and homogeneous surface, as previously shown by AFM 3D pictures of films made of the same PHBV grade [8]. As already reported in literature, a smooth surface can favor the microorganism adhesion at the material surface, thus promoting its subsequent biodegradation [36]. On the SEM image of the fracture of PHBV film (Figure 4), small elements were visible. They were boron nitride which were added in the commercial grade of PHBV as nucleating agents at the content of 1 wt %. Composite materials displayed a rougher fractured surface due to the presence ViSh fillers. No significant difference was observable between composite materials when comparing ViSh previously exhausted or not. In both cases, interfacial gaps between the matrix and the fillers were visible at high magnification, attesting that the filler/matrix interfacial adhesion was not perfect. Such defects would allow water to better diffuse in the bulk and improve accessibility to microorganisms as compared to neat PHBV. 

**Figure 5:**
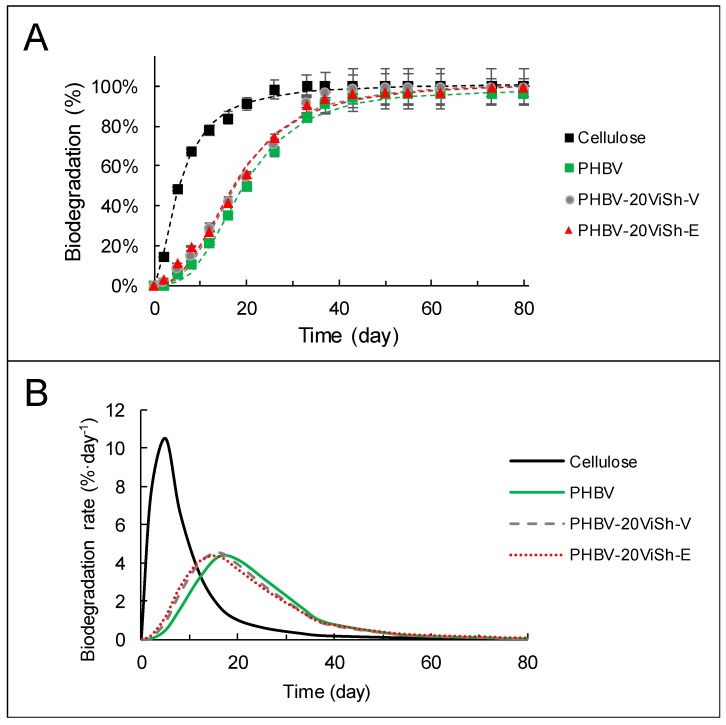
Kinetic of biodegradation (**A**) and biodegradation rate (**B**) of PHBV-based composites in soil.

The neat PHBV and PHBV/ViSh composites were subjected to respirometric tests undertaken in soil in the same aerobic conditions to those used for ViSh fillers. The resulting biodegradation curves of the composite materials are shown in Figure 5. 

No significant difference between the samples was revealed in this experiment with respect to their degradation rate. Although bacterial PHBV is known as an easily biodegradable polymer, it can be seen that its biodegradation proceeded more slowly than the cellulose reference material. However, the biodegradation curves confirmed that PHBV was fully biodegradable, with the evolution of CO_2_ reaching a plateau phase of 96 ± 5% after 55 days in soil. Even if reached later, the plateau phase of PHBV curves was similar to that of cellulose (positive control), which reflected that no additional biodegradation was expected. The rate of biodegradation of a given material being strongly dependent on soil composition, it should be always compared to that of a positive control like cellulose. In the present study, it is worth noting that PHBV exhibited a rather high rate of biodegradation. This could be explained by the milling treatment applied on composite samples, that was expected to cause easy accessibility to enzymes and microorganisms, thus increasing the available surface for degradation [18].

Even if the differences were weak, both composite materials degraded slightly better than neat PHBV, as evidenced in Figure 5B. There are different possible reasons to explain this. Firstly, it has been shown that the permeability to water vapor increases with the incorporation of natural fillers (Table 2). With the hydrolytic chain scission mechanism expected to increase with the water permeability of the materials, this could contribute to improving the biodegradation of composite materials as compared to the neat matrix. Secondly, the stiffness values of composites were higher than neat PHBV with the higher Young’s modulus determined in a previous study [18]. Material stiffness being also a factor able to promote the colonization of its surface by microoganisms [37], this could also be a reason why composites degraded faster than the neat matrix. Lastly, differences in polymer crystallinity might also lead to changes in their biodegradation patterns. However, no significant differences in crystallinity were measured between the tested materials. Generally, the amorphous phase of a polymer is more susceptible to degradation than the crystalline phase. On another note, in the case of PHBV this argument could not be evoked, since both amorphous and crystalline phases were shown to degrade at the same rate by Salomez et al. [8].

Another point that could be noted is that the difference in biodegradation observed between ViSh-V and ViSh-E was no longer visible in the PHBV-based composites. Indeed, PHBV-20ViSh-V and PHBV-20ViSh-E exhibited the same kinetics and the same final biodegradation levels (100%). This underlined that the biodegradation rate of PHBV-based composites was driven by the behavior of the PHBV matrix. 

The biodegradation curves were modeled using the Hill equation and the corresponding parameters are presented in Table 3. Deg_max_, which represents the percentage of degradation at infinite time according to the Hill equation, was close to the plateau phase observed in Figure 3 and Figure 5. Cellulose exhibited the highest maximum of biodegradation with ViSh-E and PHBV composites. It took less than 20 days for all the samples to reach 50% of Deg_max_, as indicated by k values (time when Deg = ½ Deg_max_). The constant n, representing the curve radius of the sigmoid function, was higher for PHBV-based materials, especially for neat PHBV, than for the fillers alone. This illustrated that the biodegradation started a little later for PHBV than for lignocellulosic materials, with probably a better availability and access for microorganism enzymes in the latter case. As a result, the time needed to reach the maximum of the biodegradation rate (Time_rate max_) was lower for the fillers (8 days) than for the composites materials (16 days). On the contrary, all the samples tested had similar maximum degradation rates (Deg_rate max_), with values around 4.5%∙day^−1^. 

## 3. Materials and Methods 

### 3.1. Materials

PHBV was purchased from NaturePlast (Ifs, France) under the reference PHI 002. As reported by the manufacturer, PHBV contained 1–3 mol% valerate and had a true density of 1.24 g·cm^−3^.

Vine shoots of the Syrah species were kindly provided by Jean-Michel Salmon (UEPR, INRA, Gruissan, France). They were from Gruissan, in the Languedoc-Roussillon region in the South of France. These residues displayed an initial moisture content of around 40% when they were pruned in the field. Cellulose, from pine, was supplied by Arbocel J. Rettenmaier & Söhne under the reference Arbocel® (grade BE 600-10 TG, Rosenberg, Germany) in a powder form. Particles were characterized by a cellulose content of 99.5%, a bulk density of 0.23–0.30 g·cm^−3^ (in accordance with DIN EN ISO 60), a skeletal density of 1.56 g·cm^−3^, an average thickness of 15 µm, and an average length of 18 µm (data given by the supplier).

Acetone from (99.8% of purity) and ethanol (99.9% of purity) were purchased from Biosolve Chimie (Dieuze, France) and Meridis (Montpellier, France), respectively.

### 3.2. Preparation of Vine Shoot Fillers

After pruning, the fresh ViSh were air-dried outdoors for two months. At the end of this period, the moisture content of ViSh was 20%. A first coarse milling using a shredder (AXT 22D, Bosch, Gerlingen, Germany) gave fragments of about 5 cm, that were then dried in an oven at 60 °C for 24 h. They were then milled using a cutting mill type SM 300 (Retsch, Haan, Germany) with a 4.0 mm sieve and then 2.0 mm sieve. The resulting ViSh particle size was between 1 and 0.5 mm. 

At this point, half of the ViSh were exhausted following the same extraction protocol as David et al. [38]. Batches of 10 g of substrates were introduced in Pyrex glass reagent bottles with 50 mL of acetone/water (*v*/*v*, 75:25), which were incubated at 50 °C in a shaking bath. The bottles were closed to avoid solvent evaporation. At the end of the incubation, liquid extract was separated from solid residue by centrifugation (5 min, 5000 rpm). The solid residue, noted ViSh-E, was dried under the hood overnight, then dried at 60 °C for 24 h. The second half of the ViSh were not extracted (virgin ViSh) and were kept for comparison.

Finally, exhausted ViSh and virgin ViSh particles, after being dried overnight at 60 °C, were then milled with centrifugal mill ZM 200 (Retsch, Haan, Germany) at 14,000 rpm with a 0.5 mm sieve to give ViSh-E and ViSh-V, respectively.

Finally, ViSh-V and ViSh-E, after being dried overnight at 60 °C, were then milled with centrifugal mill ZM 200 (Retsch, Haan, Germany) at 14,000 rpm with 0.5 mm sieve.

The lignin content was determined by the Klason method [39]. The first hydrolysis was done using 72% H_2_SO_4_ solution at 30 °C for 1 h and the second one was carried out using 4% H_2_SO_4_ solution at 121 °C for 1 h. Ash content was determined in triplicate from the residue after thermogravimetric analysis (Mettler TGA2, Schwerzebbach, Switzerland) at 800 °C under air. The protein content was determined from elemental analysis. Nitrogen content was multiplied by a factor of 6.25 to obtain the protein content [40]. Analytical mercaptolyses developed by Roumeas et al. [41] were conducted to determine the phenolic composition of milled vine shoots. The samples were then analyzed by a UPLC-DAD-MS system [41]. The quantification was done by UV absorbance monitoring at 280 nm.

### 3.3. Wide Angle X-Ray Diffraction (XRD)

Wide angle X-ray diffraction analysis was carried with an “in house” setup from L2C lab (Montpellier, France) to characterize the crystallinity of the fillers. ViSh powder was loaded in a capillary. A high-brightness low-power X-ray tube, coupled with aspheric multilayer optic (GeniX^3D^ from Xenocs with Cu Kα radiation λ = 1.54 Å) was used to deliver an ultralow divergent beam (0.5 mrad). The experiments were performed in transmission configuration. The scattered intensity was measured by a Schneider 2D image plate detector prototype, at a distance of 1.9 m.

### 3.4. Preparation of PHBV/ViSh Biocomposites

Biocomposites were prepared by melt mixing PHBV with virgin and exhausted vine shoot particles (20 wt %). A Brabender Plasticorder 2000 microcompounder (Brabender, Duisburg, Germany) was used. The internal temperature of the mixer was set at 180 °C, while the mixing speed was 60 rpm. The total mixing time was 6 min, whereas the mixing time after the residue addition was 3 min.

### 3.5. Scanning Electron Microscopy (SEM)

The ViSh fillers and the cross-section of PHBV-based composites were observed by scanning electron microscopy (SEM). In the case of composites, the cross-sections were obtained after cryo-fracturing in liquid nitrogen. An SEM S-4800 microscope (Hitachi, Chiyoda, Japan) was used with an acceleration voltage of 2 kV. The samples were previously coated with Pt by cathode pulverization. 

### 3.6. Biodegradation Tests

Respirometric tests were conducted in soil at 28 °C under aerobic conditions to evaluate the biodegradability of composite materials. Our assay was adapted from the US standard ASTM D5988-96, which determines aerobic biodegradation of plastic materials in soil through the released CO_2._ The release is proportional to the amount of biodegraded substrate. It provides information about the ultimate degradation step, namely the mineralization, during which the substrate is broken down into final products.

First, the composite samples were milled with a coffee grinder (DPA1 type, Moulinex, Écully, France), then sieved at 1 mm to get the same size of particles between the different samples. In the case of vine shoots, tests were directly performed using ViSh fillers without the additional milling step. The biocomposite fragments had a higher size than ViSh fillers. The carbon contents were measured by elemental analysis (ThermoQuest NA 2500, CE Instruments Ltd, Wigan, UK) for each sample. Thus, exactly 50 mg of equivalent carbon were introduced into 25 g of soil. The soil used in this study was a top soil (Verve, pH = 7.5, C/N = 39). The soil was air dried for one week, then it was sieved through a sieve of 2 mm. The dry matter content was 97%. It was determined by drying the soil at 105 °C until constant weight.

The biodegradation tests were carried out as described by Chevillard et al. [42], in hermetic glass jars (1 L, Le Parfait, Villeurbanne, France) which contained three open polypropylene vials (60 mL). One vial contained 25 g of dry soil mixed with 50 mg equivalent carbon of samples. The water content of soil was adjusted to reach 80% of the water holding capacity of the soil. The water holding capacity of soil was determined by the weight ratio of the water-saturated and dry soils. A second vial of 15 mL of NaOH (0.2 M) trapped the CO_2_ released by the microorganisms. The relative humidity was maintained at 100% inside the jar thanks to the third vial filled with distilled water. The jars were hermetically closed and incubated in the dark at 28 ± 1 °C. At the selected time, the glass jars were opened to determine the amount of CO_2_ trapped by the NaOH solution by back titration of carbonate ions. The latter were precipitated by the addition of 5 mL of barium chloride solution (20% in water), in each flask, in the presence of thymophthaleine (0.10% in ethanol), and titrated by a HCl solution (0.1 M). 

The soil-containing vials were weighted and, if necessary, the appropriate amount of water was added to stay at 80% of the soil water retention capacity. A new vial containing a NaOH solution (0.2 M) replaced the one which was just titrated. The glass jars were left open for 2 min in order to be aerated. Then, each glass jar was closed and put again in the dark at 28 °C until the next measurements.

The biodegradation tests included a control and a blank. A powder of pure cellulose (BE 600-10 TG grade) provided by Arbocel J. Rettenmaier & Söhne (Rosenberg, Germany), with a median apparent diameter (d_50_) of 18 µm, was used as control because of its well-known biodegradation pattern. For the blank, the experiment was conducted without the addition of a carbon source in the soil, to be able to measure the CO_2_ naturally produced by the soil and the CO_2_ present in the air of the glass jar. These assays were performed in triplicate. The basal blank CO_2_ production was subtracted from the measurements. The percentage of biodegradation Deg was calculated using Equation (2):

(2) $$\mathrm{Deg}\;=\;\frac{{\mathrm{CO}}_{2}\;\mathrm{material}-{\mathrm{CO}}_{2}\;\mathrm{blank}}{{\mathrm{CO}}_{2}\;\mathrm{theoretical}}\;\times 100,$$

where CO_2_ materials and CO_2_ blank are the amounts of carbon dioxide (mg) released in the test jar and in the blank control jar, respectively. CO_2_ theoretical is the theoretical amount of carbon dioxide (mg) produced by the total oxidation of the tested material. In the same way, the percentage of biodegradation of the reference material was calculated to check in the soil activity. As required by the ASTM D5988-96 standard, to check the activity of the soil and to ensure the validity of the test, cellulose (used as a positive control) should reach a biodegradation percentage higher than 70% in less than 6 months. The biodegradation curves of all the tested materials were finally normalized according to the biodegradation percentage of cellulose reached at the plateau.

The experimental degradation data were modeled with the Hill Equation (3):

(3) $$\mathrm{Deg}\;=\;\frac{{\mathrm{Deg}}_{\max}\times t^{n}}{k^{n}+{\;t}^{n}}\;,$$

where Deg at time t (day) is the percentage of degradation, Deg_max_ the percentage of degradation at infinite time, k (day) the time when Deg = ½ Deg_max_ and n the curve radius of the sigmoid degradation function.

## 4. Conclusions

Vine shoots were milled using dry fractionation to produce fine particles that were used as fillers in PHBV-based composites. The impact of ViSh fillers on the soil biodegradability of the produced composite materials was investigated according to their polyphenol contents. It was found that the incorporation of ViSh fillers in PHBV (filler content of 20 wt %) slightly accelerated the biodegradation kinetic of the composites. All the PHBV-based composites were considered fully biodegradable in soil medium, whereas ViSh-V fillers degraded only until 80% compared to the cellulose reference. ViSh-E was fully biodegraded, confirming the negative impact of polyphenols in the biodegradation mechanism. These assumptions were supported by measurements of crystallinity and SEM observations, which showed the absence of a difference in structure or morphology between particles. 

The present work displays a promising valorization pathway for vine shoots considered as an agro-residue. Their incorporation into a PHBV matrix makes it possible to design biodegradable materials. Moreover, in a biorefinery context, the extraction of polyphenols has the advantage of improving the biodegradability of vine shoots.

## Figures and Tables

**Table 1:** Biochemical composition (% dry basis) of vine shoots (ViSh) fillers.

Sample	Klason Lignin	Ashes	Tannins	Resveratrol	Proteins	C	N
ViSh-V	19.4 ± 0.5	3.9 ± 0.2	1.25 ± 0.07	0.07 ± 0.01	3.3	46.05	0.53
ViSh-E	17.7 ± 0.5	4.7 ± 0.5	0.41 ± 0.07	0.01 ± 0.00	1.7	45.96	0.27

**Table 2:** Properties of PHBV-based composites with ViSh fillers (from [18]).

	WVP (×10^13^ mol·m/(m^2^·s·Pa)	Young’s Modulus (GPa)	PHBV Crystallinity (%)
PHBV	4.3 ± 1.3	2.16 ± 0.02	55.9
PHBV-20ViSh-V	14.0 ± 1.6	2.50 ± 0.03	52.0
PHBV-20ViSh-E	21.1 ± 2.0	2.43 ± 0.04	55.4

**Table 3:** Hill parameters (Deg_max_, k, n) and related biodegradation indicators (Time_rate_ max, Deg_rate max_) of fillers and composite materials.

	Hill Parameters				Time_rate max_ (day)	Deg_rate max_ (%∙day^−1^)
	Deg_max_ (%)	k (day)	n	R^2^		
Cellulose	101 (±1)	5.5 (±0.1)	1.7 (±0.1)	0.99	5	10.5
ViSh-V	87 (±2)	11.8 (±0.6)	1.7 (±0.1)	0.98	8	4.5
ViSh-E	102 (±2)	13.9 (±0.8)	1.8 (±0.2)	0.98	8	4.3
PHBV	98 (±1)	19.1 (±0.4)	3.1 (±0.2)	0.99	16	4.3
PHBV-20ViSh-V	102 (±1)	17.6 (±0.5)	2.8 (±0.1)	0.99	16	4.4
PHBV-20ViSh-E	102 (±1)	17.3 (±0.5)	2.6 (±0.2)	0.99	16	4.5

## Abbreviations

PHBV	Poly(3-HydroxyButyrate-3-hydroxyValerate)
ViSh	Vine shoots
ViSh-V	Vine shoots that are virgin, not exhausted
ViSh-E	Vine shoots that are exhausted
